# Cellular, chromatin-accessibility, and spatial contextualization of bulk transcriptional patterns linked to positive peritoneal cytology in endometrial cancer

**DOI:** 10.3389/fgene.2026.1924936

**Published:** 2026-09-29

**Authors:** Jiangang Huang, Binglai Luo, Zhiqing Hu, Shuping Lin, Jinyong Wang, Meiyan Lin

**Affiliations:** Department of Gynecology, Longyan First Affiliated Hospital of Fujian Medical University, Longyan, Fujian, China

**Keywords:** positive peritoneal cytology, endometrial cancer, bulk RNA sequencing, single-cell RNA sequencing, chromatin accessibility, spatial transcriptomics, tumor microenvironment, qRT-PCR

## Abstract

**Background:**

Positive peritoneal cytology (PPC) identifies a clinically adverse subset of endometrial cancer, but the tumor transcriptional patterns accompanying PPC and their cellular origins remain incompletely resolved. We evaluated PPC-linked bulk RNA patterns in TCGA-UCEC and contextualized the resulting programs using independent single-cell, chromatin-accessibility, and spatial datasets.

**Methods:**

TCGA-UCEC clinical BCR XML, primary-tumor RNA sequencing, molecular annotations, ABSOLUTE purity, and TCGA Clinical Data Resource outcomes were integrated at the patient level. The final cohort contained 408 tumors with explicit PPC status. Prespecified ranked gene-set enrichment, covariate-adjusted models, multiple imputation, and survival analyses were performed. Frozen programs were mapped in GSE173682 paired scRNA-seq/scATAC-seq and GSE203612 scRNA-seq/Visium data. Seven commercial human cell models obtained from the American Type Culture Collection were used for qRT-PCR localization of representative Program A and Program B transcripts.

**Results:**

The cohort included 351 PPC-negative and 57 PPC-positive tumors. No individual gene reached genome-wide BH-FDR<0.05. Ranked analysis showed relative enrichment of epithelial–mesenchymal transition (EMT), hypoxia, TGF-beta signaling, and angiogenesis in PPC-positive tumors, with relative depletion of cytotoxic-lymphocyte, T-cell-inflamed, and antigen-presentation programs. PPC remained associated with shorter progression-free interval (PFI) (HR = 2.77, 95% CI = 1.73–4.43), overall survival (OS) (HR = 2.84, 95% CI = 1.66–4.84), and disease-specific survival (DSS) (HR = 3.47, 95% CI = 1.86–6.46). Program A showed a replicated broad stromal-vascular source, whereas Program B localized to NK/CD8 compartments. After cell-type and patient adjustment, only Program B retained clear RNA-ATAC concordance. Program A was strongly spatially organized in one UCEC section (Moran’s I = 0.769; FDR = 0.0015), but composition-adjusted analyses did not support cytotoxic exclusion or stable hypoxia/TGF-beta coupling. qRT-PCR showed higher Program A scores in stromal/perivascular models than in tumor-epithelial models (mean difference 1.52 standardized units, 95% CI = 1.34–1.71; P < 0.001) and higher Program B scores in NK than in non-cytotoxic models (mean difference 2.09, 95% CI = 1.97–2.22; P < 0.001). Tumor CD274 transcript abundance was not higher in PPC-positive tumors and did not credibly modify PPC-associated risk.

**Conclusion:**

PPC-positive endometrial cancers exhibit coordinated stromal/mesenchymal-high and cytotoxic/antigen-presentation-low ranked bulk transcriptional directions. Single-cell, chromatin-accessibility, spatial, and qRT-PCR data consistently localize the dominant programs to stromal–vascular and cytotoxic-lymphocyte compartments. PPC remains a strong adverse clinical marker, whereas tumor CD274 transcript abundance does not explain its prognostic association.

## 1 Introduction

Endometrial cancer is among the most common gynecological malignancies worldwide, and its incidence continues to increase in parallel with aging populations and metabolic risk factors (Sung et al., 2021). Contemporary molecular classification has refined the traditional histopathological framework by identifying POLE-ultramutated, mismatch-repair-deficient, copy-number-low, and copy-number-high groups with distinct biological and clinical behavior (The Cancer Genome Atlas Research Network et al., 2013). Even within these molecularly informed strata, readily available surgical and pathological variables continue to carry prognostic information that is not fully captured by the tumor stage alone.

Peritoneal washing cytology is one such variable. Positive peritoneal cytology (PPC) was removed from formal FIGO staging in 2009, yet systematic reviews and large retrospective studies continue to associate it with recurrence and inferior survival, including in otherwise early-stage disease (Pergialiotis et al., 2024; Fegus et al., 2024; Seagle et al., 2018; Kanno et al., 2022). This persistent signal has renewed interest in whether PPC merely marks greater anatomical tumor burden or also reflects a distinct tumor state. The distinction matters: a biomarker that is only a surrogate of advanced disease may add little biological information, whereas a reproducible transcriptional pattern could help explain why PPC-positive patients remain at elevated risk after conventional clinicopathological adjustment.

Bulk tumor RNA sequencing is well suited to patient-level clinical association, but it combines malignant, immune, vascular, and stromal transcripts. Consequently, a bulk signal can arise from changes in cell abundance, from altered transcription within a cell population, or from both. Single-cell RNA sequencing can identify the cellular compartments contributing to a program, while single-cell chromatin-accessibility data can determine whether the same compartments show concordant gene-activity patterns. Spatial transcriptomics adds a complementary tissue-level view by evaluating whether a program is spatially organized. These modalities are especially useful when treated as contextual layers rather than as replacements for a clinically labeled cohort. GSE173682 provides matched scRNA-seq and scATAC-seq profiles from human gynecological tumors (Regner et al., 2021), and GSE203612 includes UCEC single-cell profiles and a matched Visium section within a pan-cancer atlas (Barkley et al., 2022).

We therefore designed a patient-anchored, program-centered study. TCGA-UCEC was the only dataset used to estimate PPC-associated effects. We first evaluated a prespecified CD274 hypothesis and ranked transcriptional programs in the PPC-labeled cohort. We then froze three biological programs: Program A, representing an ECM/CAF–mesenchymal–vascular pattern; Program B, representing cytotoxic T/NK transcription; and Program C, representing IFN/antigen presentation. Independent single-cell, chromatin-accessibility, and spatial datasets were used to determine where these programs were expressed and how they were organized. Commercial ATCC cell models were subsequently used for qRT-PCR assessment of Program A and Program B cellular localization. The study was structured to distinguish PPC-linked clinical evidence from multimodal contextual evidence and to avoid causal or treatment-response claims not supported by the data.

## 2 Materials and methods

### 2.1 Study design and inference framework

This study combined a retrospective secondary analysis of de-identified public data with an orthogonal commercial-cell qRT-PCR experiment. The inferential hierarchy was fixed before the multimodal analyses. TCGA-UCEC was the only cohort containing patient-level PPC status and therefore the only dataset used to estimate PPC-associated clinical or transcriptional effects. GSE173682 and GSE203612 were used solely for cellular, chromatin-accessibility, and within-section spatial contextualization. Cells, modality samples, and spatial spots were not treated as independent patients. All program definitions, scoring methods, FDR families, biological-replicate units, and stopping rules were frozen in a version-controlled manifest before downstream result inspection.

### 2.2 TCGA-UCEC cohort construction

Clinical BCR XML files, primary-tumor STAR-count RNA-seq data, TCGA Pan-Cancer molecular resources, TCGA Clinical Data Resource outcomes, and ABSOLUTE tumor-purity estimates were integrated using the TCGA patient barcode. The TCGA Clinical Data Resource provided standardized progression-free interval (PFI), overall survival (OS), and disease-specific survival (DSS) endpoints (Liu et al., 2018). Of 548 clinical XML cases, 411 had an explicit positive or negative peritoneal-cytology result. Three PPC-labeled cases had no usable primary-tumor RNA-seq, leaving 408 patients: 351 PPC-negative and 57 PPC-positive. Only sample-type 01 primary tumors were retained. When more than one RNA file was available for a patient, the 01A aliquot was preferred, and the library with the largest total read count was selected. This selection did not use PPC, CD274, or outcome information.

Clinical covariates included age at diagnosis, histological type, tumor grade, myometrial invasion, lymph-node status, residual tumor, surgical approach, four-class TCGA molecular subtype, tumor mutational burden, and ABSOLUTE tumor purity. FIGO stage was displayed descriptively and evaluated in sensitivity analyses. The primary adjustment set used the component clinicopathological and molecular variables to reduce the risk of conditioning on a composite stage variable that may partly incorporate downstream information. Missing covariates were multiply imputed in 20 datasets using chained equations (van Buuren and Groothuis-Oudshoorn, 2011).

### 2.3 Frozen biological programs

Program definitions were frozen after a negative candidate-screening workflow. A signed weighted gene co-expression network was constructed without using PPC labels (Langfelder and Horvath, 2008). Leading-edge genes from seven prespecified FDR-significant ranked gene sets were then evaluated through sequential PPC-association, robustness, module-membership, survival, and immune-correlation layers. None of 246 candidates passed the first three layers or all four layers; therefore, no single hub gene was promoted. Program A comprised 29 genes defined as the intersection of the red ECM/CAF–mesenchymal module with epithelial–mesenchymal transition (EMT) or angiogenesis leading-edge genes. Program B comprised CCL5, CD8A, CD8B, CTSW, GNLY, GZMA, GZMB, GZMH, NKG7, and PRF1. Program C comprised B2M, HLA-A, HLA-B, HLA-C, NLRC5, PSMB8, PSMB9, TAP1, TAP2, and TAPBP. Hallmark hypoxia and Hallmark TGF-beta signaling were retained as companion states. CD274 was a prespecified negative reference and was not included in any program.

### 2.4 Bulk RNA analysis and patient-level scoring

Tumor CD274 transcript abundance was expressed as log2(TPM+1). The primary comparison used a multiply imputed linear model with PPC as the exposure and the frozen clinicopathological and molecular covariates. Unadjusted, complete-case, stage-adjusted, and restriction-based sensitivity models were also evaluated. Genome-wide transcriptional effects were estimated using limma-voom (Law et al., 2014), with the same prespecified adjustment structure where estimable. Two-sided P-values were corrected using the Benjamini–Hochberg procedure (Benjamini and Hochberg, 1995). Ranked gene-set enrichment analysis used the complete moderated-statistic ordering rather than a list of selected genes (Subramanian et al., 2005). Positive normalized enrichment scores indicated relative enrichment in PPC-positive tumors; negative values indicated relative depletion in PPC-positive tumors. Patient-level Program A, B, and C scores were calculated using singscore (Foroutan et al., 2018).

### 2.5 Clinical-outcome analysis

Kaplan–Meier curves were used for unadjusted PFI and OS visualization (Kaplan and Meier, 1958). Cox proportional-hazards models estimated adjusted PPC effects for PFI, OS, and DSS (Cox, 1972). Tumor CD274 transcript abundance entered the main models as a continuous standardized variable. PPC-by-CD274 interaction terms were evaluated without dichotomizing CD274 or selecting an outcome-optimized cut point. Proportional-hazards assumptions were assessed using Schoenfeld residuals. DSS was interpreted as a supporting endpoint because only 48 DSS events were observed.

### 2.6 Single-cell RNA-seq datasets and quality control

GSE173682 contributed five primary or non-metastatic endometrial tumors with paired scRNA-seq and scATAC-seq modalities (Regner et al., 2021). GSE203612 contributed UCEC1–UCEC3 scRNA-seq profiles and a UCEC3 Visium section (Barkley et al., 2022). The two scRNA-seq datasets were processed separately and were not merged into a common expression matrix. Each technical library underwent independent quality control. Cells with fewer than 200 detected genes or mitochondrial-read fractions of at least 20% were removed; sample-specific upper thresholds for gene and count distributions were set using robust median-plus-five-MAD rules. Doublets were detected within the sample using scDblFinder (Germain et al., 2021). Harmony was used only to facilitate visualization and annotation, not as the expression scale for program statistics (Korsunsky et al., 2019).

Level-1 cell annotation was completed and frozen before program scores were viewed. The common vocabulary included malignant-like epithelial, fibroblast/CAF, pericyte/vascular smooth-muscle, endothelial, myeloid, CD8 T, CD4/Treg, NK, B/plasma, and mast cells, with the non-malignant epithelial category retained only when supported. Annotation used program-excluded canonical markers, source labels, reference mapping, and copy-number evidence where available. Epithelial cells were conservatively termed malignant-like when copy-number support was incomplete.

### 2.7 Single-cell program scoring and source contrasts

Program scores were calculated using UCell on QC-passed, non-integrated count matrices using a frozen maximum rank of 1,500 (Andreatta and Carmona, 2021). Continuous UCell scores were primary. The fraction of cells above the dataset-wide 75th percentile was a distributional sensitivity metric and was not interpreted as biological positivity. The biological replicate was the patient-by-cell-type unit; cell types represented by fewer than 20 cells in a patient were excluded from formal contrasts. Program A source contrasts compared broad stromal compartments with other cells, and Program B contrasts compared CD8/NK with other immune cells. GSE173682 estimates were derived from patient-level models with confidence intervals and FDR correction. Because GSE203612 contained only three UCEC patients and two effective patients for some stromal contrasts, medians and patient-level ranges were reported without formal P-values.

### 2.8 scATAC-seq analysis

The GSE173682 scATAC-seq modality was analyzed with Signac using GENCODE release 47 on GRCh38.p14 (Stuart et al., 2021). Cells underwent fragment, transcription-start-site enrichment, nucleosome-signal, and doublet quality control. RNA-derived cell labels were transferred across separately measured modalities; these data were patient-matched but were not same-cell multiome measurements. GeneActivity matrices used a 2-kb upstream extension and were scored using UCell. Program coverage was audited after annotation harmonization. ChromVAR deviation scores were calculated for frozen transcription-factor motif families (Schep et al., 2017). Motif-program associations were evaluated first across cell types and then after adjustment for frozen cell type and patient. Residual RNA-ATAC concordance was summarized with Spearman correlations and patient-cluster bootstrap confidence intervals. Aggregate Tn5 profiles around strand-aware transcription start sites were treated as accessibility context, rather than as promoter activation or proof of direct regulation.

### 2.9 Spatial transcriptomic analysis

The UCEC3 Visium section from GSE203612 was aligned to its real high-resolution H&E image. No tumor-core, interface, fibrotic-stroma, or necrosis polygons were invented because no pathologist-frozen regions were available. Robust cell type decomposition (RCTD) decomposed spot-level expression using the matched uterine corpus endometrial carcinoma (UCEC) single-cell reference (Cable et al., 2022). Program A, B, and C UCell scores were calculated on the Visium expression matrix. Spatial organization was evaluated with Moran’s I and 1,000 block permutations. A prespecified sensitivity analysis removed all 29 Program A genes from both reference and query before repeating RCTD to assess direct gene-reuse circularity. For the Program A-cytotoxic relationship, analyses proceeded from raw spatial-lag correlation to composition-aware centered log-ratio (CLR) balance and then to residual models adjusting for tumor fraction, stromal–vascular fraction, unique molecular identifier (UMI) depth, and detected genes. Hypoxia and TGF-beta associations were analyzed using the same framework. Because only one UCEC section was available, all spatial inference was explicitly within-section.

### 2.10 Immune and stromal deconvolution in TCGA

Bulk immune and stromal enrichment was assessed using xCell (Aran et al., 2017) and MCP-counter (Becht et al., 2016). The prespecified interpretation required directional agreement and FDR support across both methods for a strong replication claim. Estimates that were significant in only one algorithm were reported as single-method findings. ABSOLUTE tumor purity was examined as a negative-control tissue-composition variable.

### 2.11 Commercial cell culture and qRT-PCR

Commercial human cells were obtained from the American Type Culture Collection (ATCC): HEC-1-A endometrial adenocarcinoma cells (HTB-112), KLE endometrial adenocarcinoma cells (CRL-1622), T-HESC endometrial stromal cells (CRL-4003), primary uterine smooth-muscle cells (PCS-460-010), EA.hy926 endothelial cells (CRL-2922), NK-92 cells (CRL-2407), and NK-92MI cells (CRL-2408). Cells were maintained according to ATCC recommendations at 37 °C in a humidified atmosphere containing 5% CO2 and were confirmed to be free of *mycoplasma* before RNA collection. NK-92 cells were maintained as an IL-2-dependent model and NK-92MI as its IL-2-independent derivative; both are established cytotoxic NK models (Gong et al., 1994).

Six Program A transcripts (LUM, COL3A1, MGP, ACTA2, PDGFRB, and BGN) and six Program B transcripts (NKG7, GNLY, PRF1, GZMB, CTSW, and CCL5) were selected before qRT-PCR analysis. Five independent culture experiments were performed for each cell model, with technical triplicates for each assay. Total RNA was isolated from each independent culture, reverse-transcribed, and quantified using SYBR Green qRT-PCR. RPLP0 and HPRT1 were used as reference genes and combined geometrically for normalization. Primer efficiency, melt-curve specificity, no-template controls, and no-reverse-transcription controls were assessed in accordance with MIQE principles (Bustin et al., 2009). Statistical analyses were based on −Delta Ct; 2^−ΔΔCt^ was used for descriptive fold-change presentation (Livak and Schmittgen, 2001). Each program score was calculated as the unweighted mean of gene-wise standardized -Delta Ct values. Gene-level P-values were corrected separately within Program A and Program B.

### 2.12 Statistical analysis

All tests were two-sided. Unless a separate prespecified family was defined, multiplicity was controlled at BH-FDR<0.05 (Benjamini and Hochberg, 1995). Standardized beta coefficients, hazard ratios, 95% confidence intervals, and exact P-values are reported where appropriate. The biological replicate was the patient for TCGA, the patient-by-cell-type unit for single-cell and scATAC source contrasts, and the tissue section or spatial block for within-section spatial analyses. Technical libraries, single cells, and Visium spots were not treated as independent patients. Analyses were performed in R 4.5.1 using the frozen software versions documented in the reproducibility manifest.

## 3 Results

### 3.1 PPC-labeled TCGA cohort and clinical context

The audited clinical XML contained 548 TCGA-UCEC cases. Explicit positive or negative cytology results were available for 411 cases; three lacked a usable primary-tumor RNA-seq file. The final patient-level cohort therefore included 408 patients, comprising 351 PPC-negative and 57 PPC-positive tumors (Figure 1A). Molecular subtype data were available for 380 patients, and ABSOLUTE purity data were available for 397.

**FIGURE 1:**
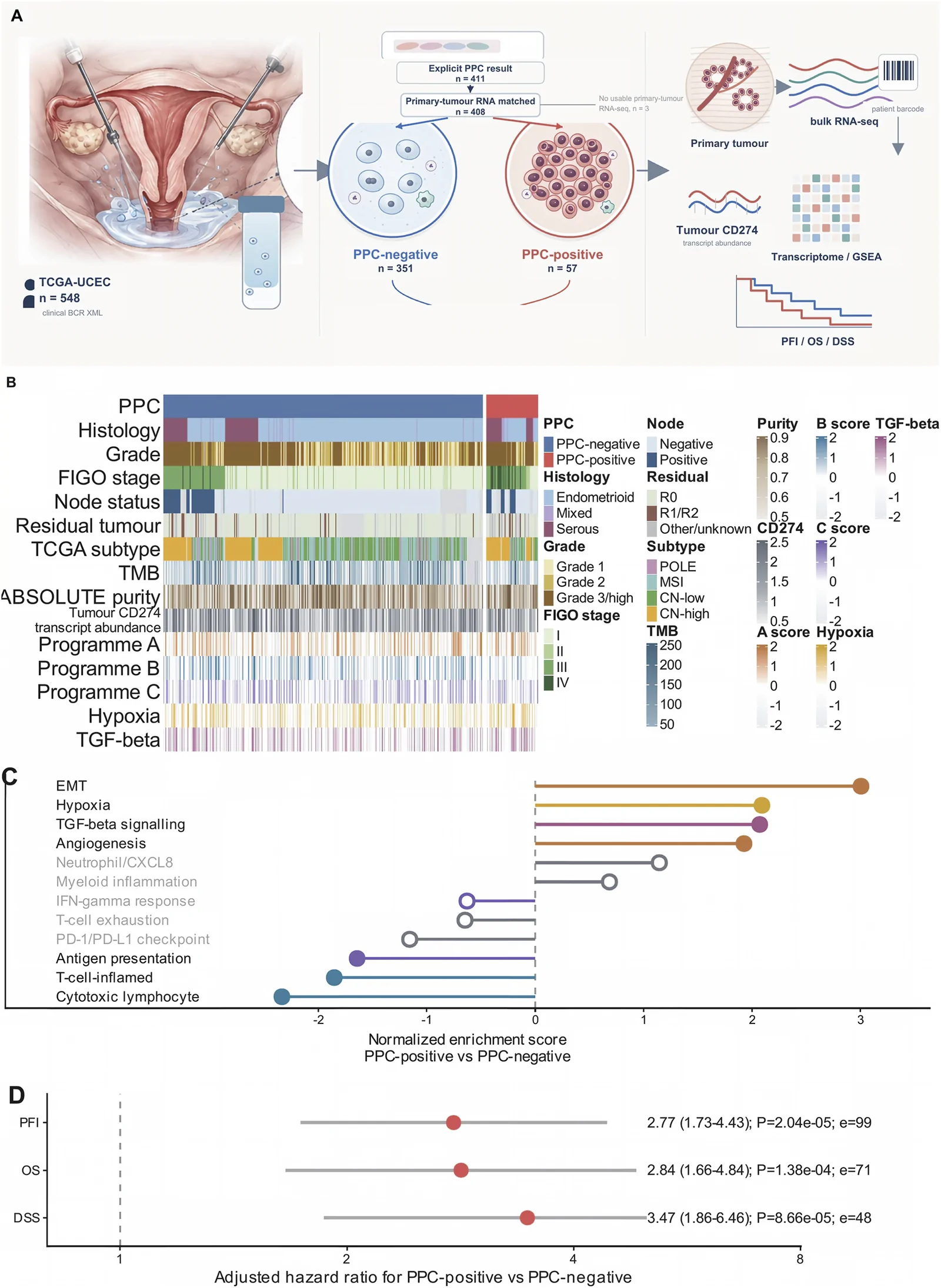
TCGA clinical anchor and ranked bulk transcriptional patterns. **(A)** Audited cohort construction from 548 clinical XML cases to 408 primary-tumor RNA-matched patients with explicit PPC status. **(B)** Patient-level clinical, molecular, CD274, and frozen-program heatmap; the display is descriptive. **(C)** Prespecified ranked GSEA. Positive NES indicates relative enrichment in PPC-positive tumors and negative NES relative depletion. No individual gene reached genome-wide BH-FDR<0.05. **(D)** Adjusted PPC effects for PFI, OS, and DSS. HR > 1 indicates higher risks in PPC-positive patients.

PPC-positive tumors were disproportionately represented in high-risk clinical and molecular categories (Figure 1B). Serous histology occurred in 42.1% of PPC-positive and 17.7% of PPC-negative patients, grade 3/high disease in 73.7% and 56.4%, node-positive disease in 34.8% and 16.6% of patients with evaluable nodes, and FIGO III–IV disease in 77.2% and 19.4%, respectively. Copy-number-high tumors accounted for 58.5% of molecularly classified PPC-positive tumors and 26.3% of PPC-negative tumors. These imbalances reinforced the need for multivariable adjustment and precluded interpreting PPC as an isolated biological exposure.

### 3.2 Ranked bulk transcriptional patterns and prognosis

No individual gene reached BH-FDR<0.05 among 38,021 tested genes. Nevertheless, ranked GSEA identified coordinated directions at the program level (Figure 1C). EMT showed the strongest relative enrichment in PPC-positive tumors (NES = 3.006; FDR = 7.753 × 10^−27^), followed by hypoxia (NES = 2.091; FDR = 2.111 × 10^−9^), TGF-beta signaling (NES = 2.071; FDR = 1.331 × 10^−4^), and angiogenesis (NES = 1.926; FDR = 0.00165). Cytotoxic-lymphocyte transcription was relatively depleted (NES = −2.336; FDR = 5.634 × 10^−7^), as were the T-cell-inflamed signature (NES = −1.854; FDR = 0.00857) and antigen processing/presentation (NES = −1.643; FDR 0.0282). These findings represented coordinated rank shifts rather than conventional differentially expressed genes.

PPC remained strongly associated with adverse outcomes after adjustment for the frozen clinicopathological and molecular covariates (Figure 1D). The adjusted HR was 2.77 for PFI (95% CI = 1.73–4.43; P = 2.04 × 10^−5^; 99 events), 2.84 for OS (95% CI = 1.66–4.84; P = 1.38 × 10^−4^; 71 events), and 3.47 for DSS (95% CI = 1.86–6.46; P = 8.66 × 10^−5^; 48 events). Because PPC-positive patients had substantially more advanced and aggressive disease, these estimates were interpreted as adjusted observational associations rather than causal effects.

### 3.3 Single-cell localization of the frozen programs

After per-sample quality control and doublet removal, GSE173682 contributed 29,964 singlets from five endometrial-tumor patients and GSE203612 contributed 5,648 singlets from three UCEC patients (Figures 2A,B). Program A showed a broad stromal source in both datasets (Figure 2C). Its highest median source was pericyte/vascular smooth-muscle cells in GSE173682 and fibroblast/CAF cells in GSE203612, indicating cross-dataset agreement at the compartment level but heterogeneity at the finer stromal-subtype level. Program B was highest in NK and CD8 T compartments in both datasets. Program C localized primarily to NK cells in GSE173682 but to B/plasma cells in GSE203612, and its prespecified epithelial/myeloid/endothelial contrast was unsupported.

**FIGURE 2:**
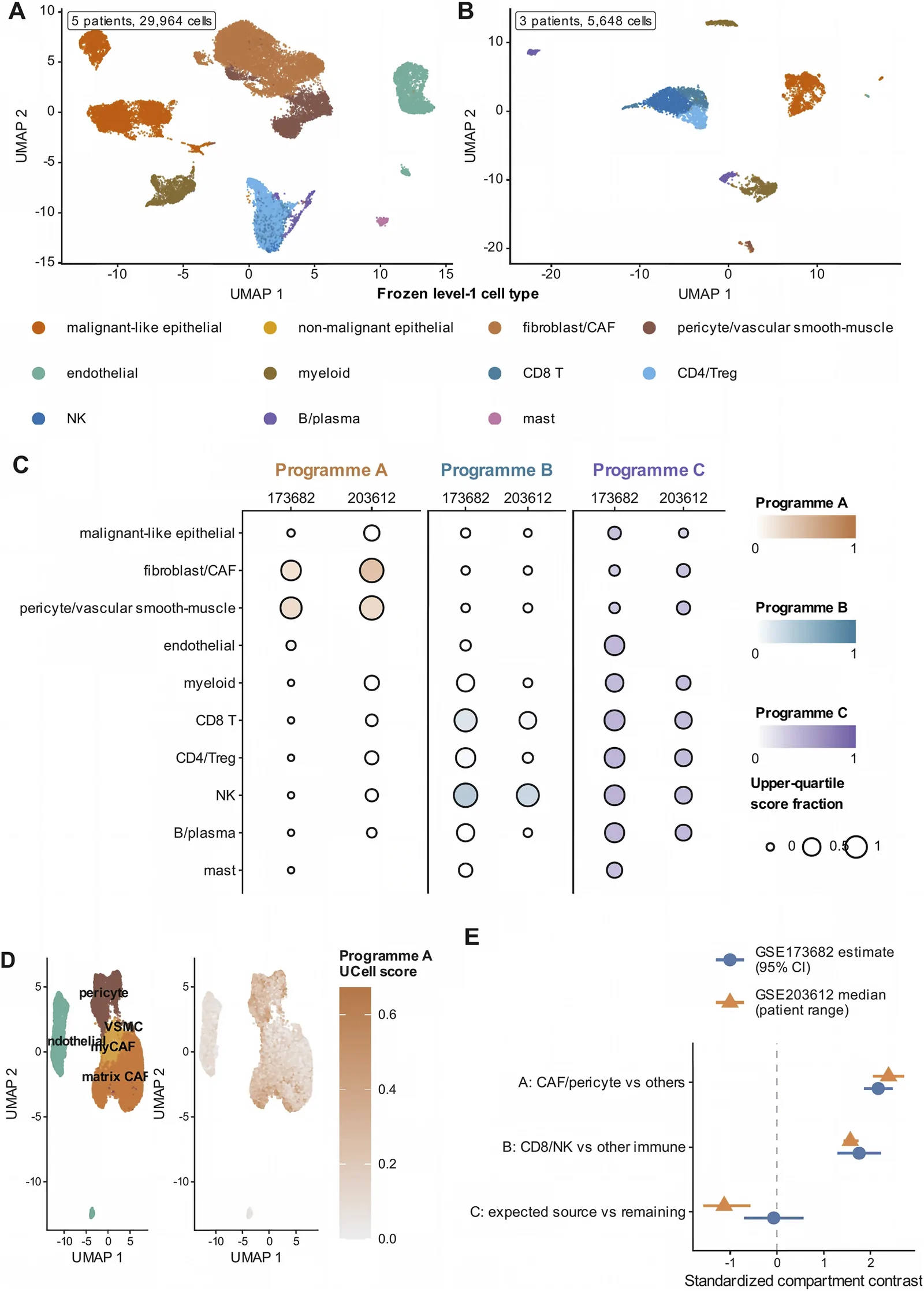
Single-cell cellular contextualization. **(A)** GSE173682 level-1 atlas of 29,964 QC-passed singlets from five patients. **(B)** GSE203612 atlas of 5,648 singlets from three UCEC patients. **(C)** Program A/B/C cellular-source matrix; the fraction above the dataset-wide 75th percentile is a distributional metric, not biological positivity. **(D)** Fine stromal analysis of Program A. **(E)** Cross-dataset source contrasts. GSE173682 circles show model estimates and 95% CIs; GSE203612 triangles show medians and patient-level ranges.

Fine stromal re-clustering in GSE173682 included 16,745 fibroblast/CAF, pericyte/vascular smooth-muscle, and endothelial cells from five patients (Figure 2D). Pericytes had the highest median Program A UCell score (0.252) and the largest fraction above the dataset-wide upper-quartile threshold (0.761). Matrix cancer-associated fibroblast (CAF), myofibroblastic CAF, vascular smooth-muscle-like, and endothelial groups also contributed to the program, supporting the term stromal–vascular rather than pericyte-specific or CAF-specific.

Patient-level source contrasts confirmed these patterns (Figure 2E). In GSE173682, the standardized Program A stromal contrast was 2.168 (95% CI = 1.860–2.476; FDR = 6.03 × 10^−17^), with a directionally concordant median beta of 2.388 in GSE203612. The Program B CD8/NK contrast was 1.760 (95% CI = 1.297–2.222; FDR 1.47 × 10^−7^) in GSE173682 and directionally 1.571 in GSE203612. The Program C expected-source contrast was near zero in GSE173682 and inverse in GSE203612, providing no replicated source localization.

### 3.4 Chromatin-accessibility context after controlling for cell identity

The scATAC analysis retained 30,755 singlets from the same five GSE173682 patients (Figure 3A). GeneActivity coverage was 27 of 29 Program A genes and 10 of 10 genes for Programs B and C. Program A and Program B GeneActivity localized to the same broad source compartments identified using scRNA-seq, whereas Program C again showed an opposite or inconsistent expected-source contrast (Figure 3B).

**FIGURE 3:**
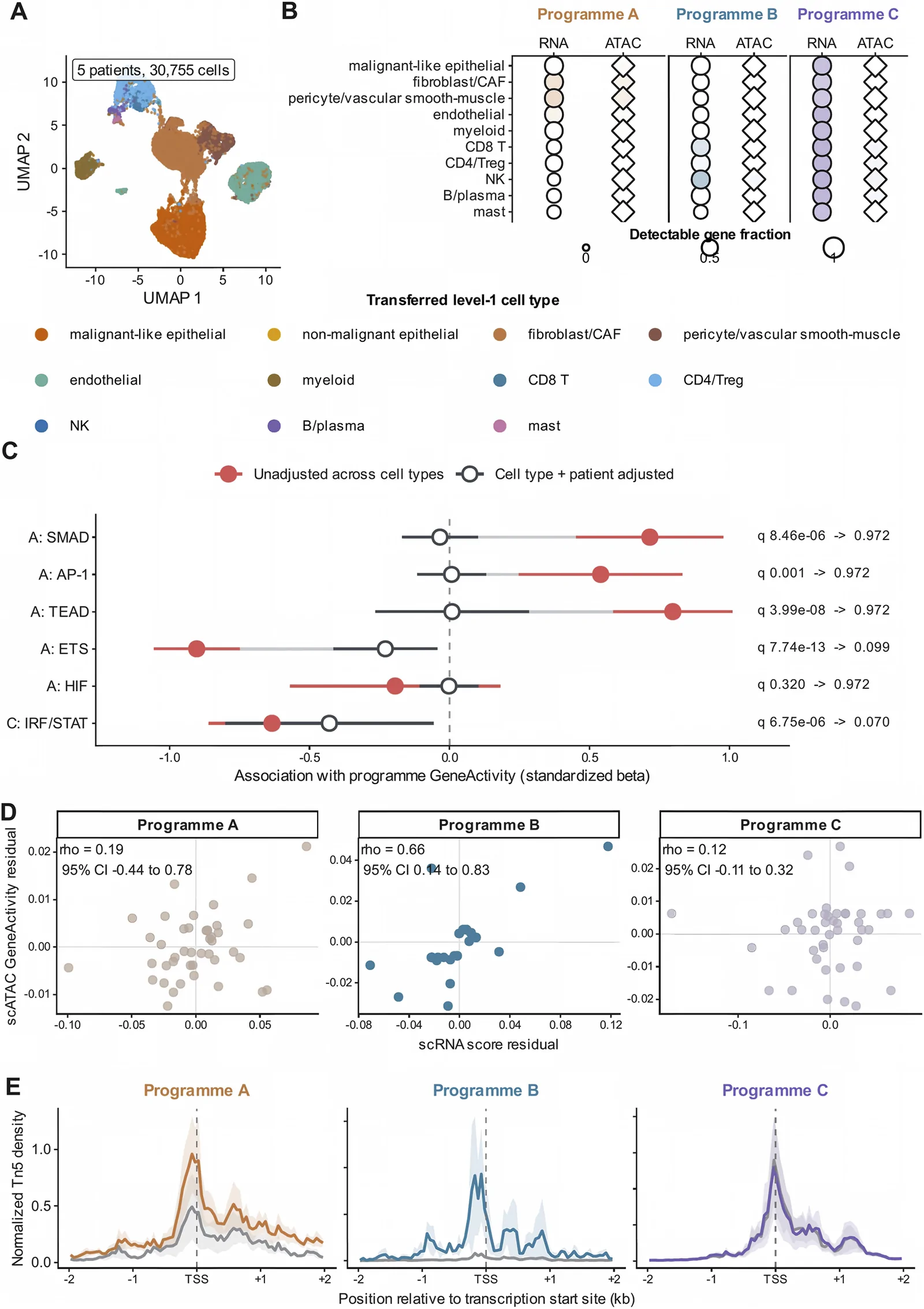
scATAC cell identity and residual chromatin-accessibility context. **(A)** scATAC atlas of 30,755 singlets from five patient-matched samples; label transfer is cross-modality integration, rather than same-cell multiome measurement. **(B)** RNA and GeneActivity program localization. **(C)** Motif-program associations before and after cell-type/patient adjustment; adjusted associations did not pass BH-FDR<0.05. **(D)** Cell-type- and patient-residualized RNA-ATAC concordance. **(E)** Program-level Tn5 profiles around transcription start sites, shown as accessibility context rather than promoter activation or direct regulation.

Across cell types, Program A GeneActivity initially showed strong associations with suppressor of mothers against decapentaplegic (SMAD), AP-1, and TEA domain (TEAD) motif activity while showing an inverse association with E26 transformation-specific (ETS) activity. However, all displayed associations attenuated after adjustment for frozen cell type and patient (Figure 3C). For example, the A-SMAD beta changed from 0.715 (q = 8.46 × 10^−6^) to −0.034 (q = 0.972), A-AP-1 from 0.539 (q = 0.00110) to 0.0074 (q = 0.972), and A-TEAD from 0.797 (q = 3.99 × 10^−8^) to 0.0090 (q = 0.972). These reductions indicated that the unadjusted associations were largely explained by cell identity, rather than by a stable within-compartment regulatory relationship.

Residualized RNA-ATAC concordance supported the same interpretation (Figure 3D). Program A had a Spearman rho of 0.194 (patient-bootstrap 95% CI = −0.435 to 0.780), and Program C had a rho of 0.122 (95% CI = −0.110 to 0.321). Program B alone retained a clearly positive residual association (rho 0.660; 95% CI 0.142–0.830). Aggregate promoter Tn5 curves were higher in the expected source compartments for Programs A and B, whereas Program C source and comparator curves overlapped (Figure 3E). These curves were treated as descriptive accessibility context and not as evidence of promoter activation or direct transcriptional regulation.

### 3.5 Within-section spatial organization

The real UCEC3 H&E section and 1,243 graph-connected, QC-passed spots were used without invented pathology polygons (Figure 4A). Among 1,244 spots with RCTD estimates, 1,227 were tumor-epithelial-dominant, 14 vascular-dominant, and 2 CAF/stroma-dominant; no spot was T/NK-dominant. Continuous mean weights, nevertheless, included tumor epithelium 80.78%, vascular cells 7.60%, CAF/stroma 5.64%, and T/NK cells 2.51% (Figure 4B).

**FIGURE 4:**
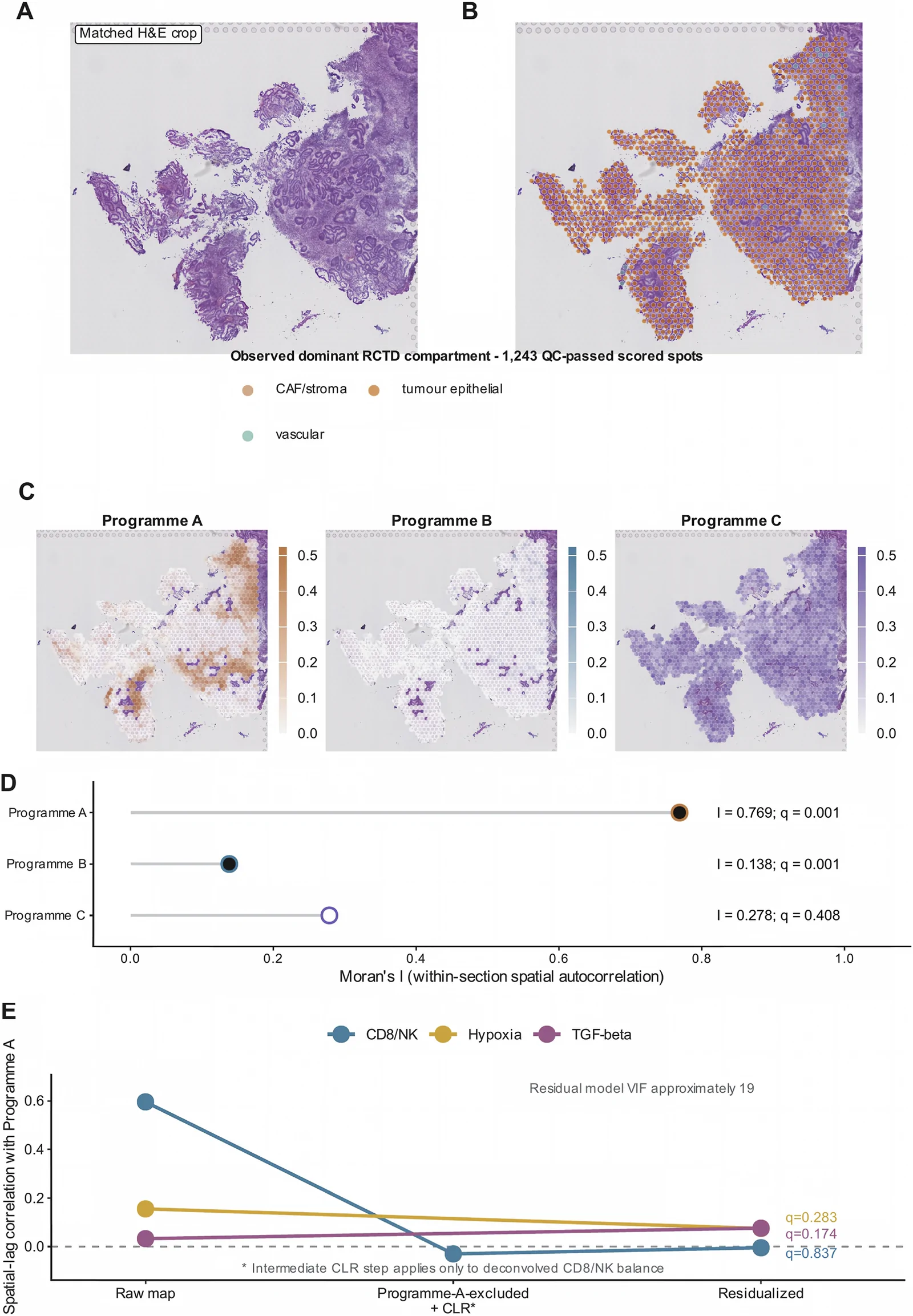
H&E image obtained from the publicly available GSE203612 dataset (Barkley et al., 2022). **(A)** Real H&E image without invented pathological regions. **(B)** RCTD dominant-compartment overlay. **(C)** Program A/B/C spatial maps. **(D)** Moran’s I estimates and permutation FDR. **(E)** Stepwise diagnostic of Program A spatial associations: raw, Program-A-gene-excluded, composition-aware, and technically residualized relationships. The available section did not support cytotoxic exclusion or stable hypoxia/TGF-beta coupling.

Program A showed strong spatial organization (Moran’s I = 0.769; FDR = 0.00150), whereas Program B showed weak but statistically detectable organization (I = 0.138; FDR = 0.00150). Program C did not pass the prespecified gate (I = 0.278; FDR = 0.408) (Figures 4C,D).

The apparent Program A-cytotoxic relationship changed substantially after composition handling (Figure 4E). The raw A-CD8/NK spatial-lag correlation was 0.596 and remained 0.601 after excluding all 29 Program A genes from the RCTD reference and query. After CLR composition handling, the correlation decreased to −0.030, and after residualization for tumor fraction, stromal–vascular fraction, UMI depth, and detected genes, it was −0.0048 (FDR = 0.837). Residual Program A-hypoxia and Program A-TGF-beta correlations were 0.0749 (FDR = 0.283) and 0.0760 (FDR = 0.174), respectively. Thus, the available section supported spatial organization of Program A but did not provide evidence for cytotoxic exclusion or stable hypoxia/TGF-beta coupling. The residual model was collinearity-limited, with variance-inflation factors of approximately 19, and was interpreted cautiously.

### 3.6 Tumor CD274 transcript abundance did not explain the PPC association

Tumor CD274 transcript abundance was slightly lower, rather than higher, in PPC-positive tumors, but the effect was small and imprecise (Figure 5A). The Wilcoxon P-value was 0.392, and Hedges g was −0.09. In the multiply imputed primary model, the PPC-positive-minus-PPC-negative beta was −0.031 log2 (TPM+1) units (95% CI = −0.234 to 0.172; P = 0.765). Every complete-case, stage-adjusted, and restriction-based sensitivity interval crossed zero (Figure 5B).

**FIGURE 5:**
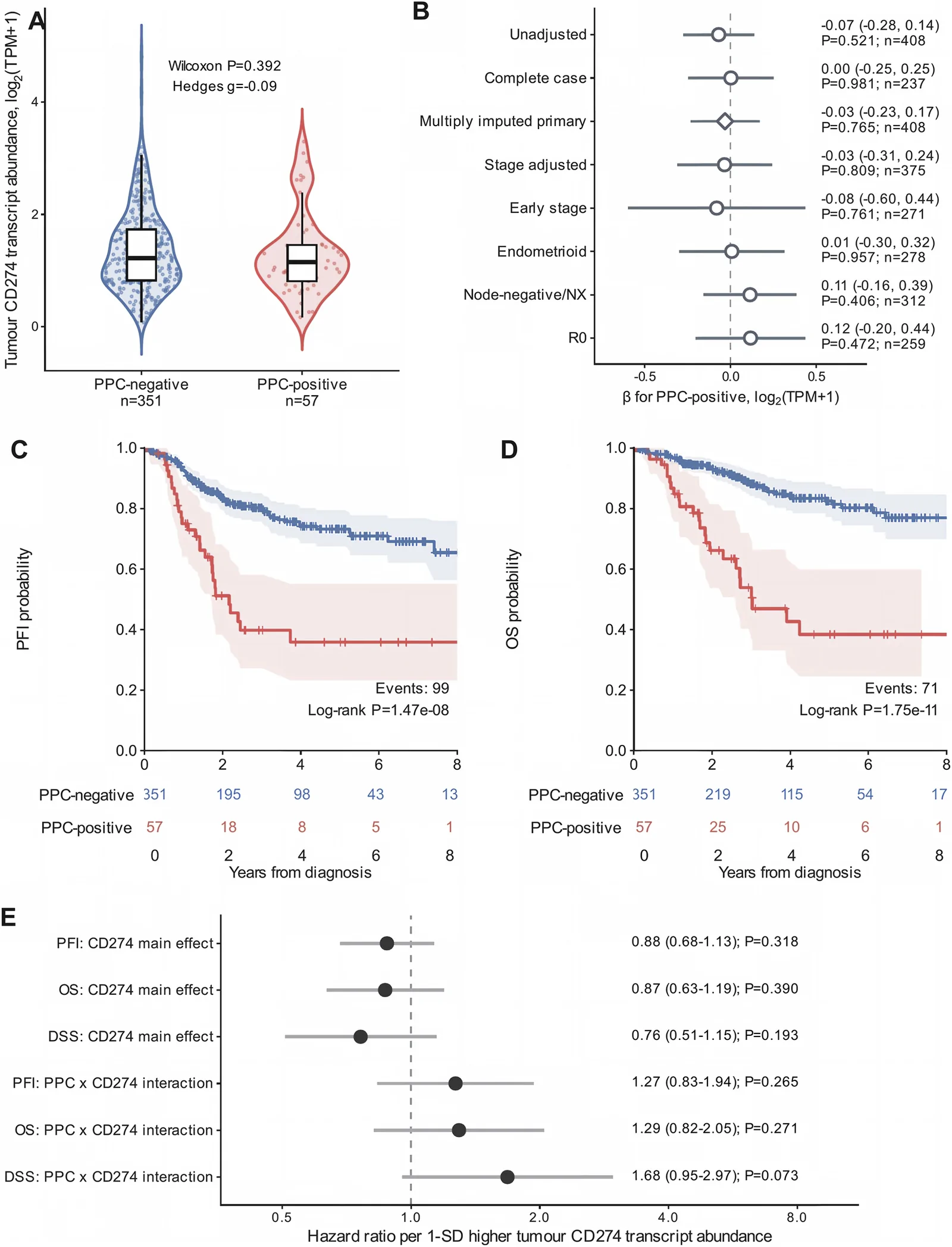
Tumor CD274 transcript abundance and clinical outcomes. **(A)** Observed tumor CD274 transcript abundance by PPC status. **(B)** Primary and sensitivity model estimates; every confidence interval crossed zero. **(C,D)** Unadjusted PFI and OS Kaplan–Meier curves with risk tables. **(E)** Continuous CD274 main effects and PPC-by-CD274 interaction terms for PFI, OS, and DSS. The endpoint is tumor CD274 transcript abundance, rather than PD-L1 immunohistochemistry, TPS, or CPS.

Unadjusted Kaplan–Meier curves showed substantially shorter PFI and OS in the PPC-positive group (Figures 5C,D), consistent with the adjusted estimates reported above. Tumor CD274 transcript abundance itself was not associated with PFI (HR per 1 SD = 0.879; 95% CI = 0.681–1.133; P = 0.318), OS (HR = 0.870; 95% CI = 0.634–1.194; P = 0.390), or DSS (HR = 0.763; 95% CI = 0.507–1.147; P = 0.193). PPC-by-CD274 interaction estimates also crossed the null for PFI, OS, and DSS (Figure 5E). The DSS interaction was numerically larger but remained uncertain (HR = 1.681; 95% CI = 0.952–2.968; P = 0.0735) and was not interpreted as effect modification.

### 3.7 qRT-PCR localization across ATCC cell models

Seven ATCC cell models represented tumor epithelial, stromal, perivascular/smooth-muscle, endothelial, and cytotoxic-lymphocyte compartments. The Program A heatmap showed higher expression of LUM, COL3A1, MGP, ACTA2, PDGFRB, and BGN in T-HESC and uterine smooth-muscle cells than in HEC-1-A and KLE tumor cells, with intermediate expression in EA.hy926 endothelial cells (Figure 6A). The unweighted Program A qRT-PCR score was 1.52 standardized units higher in stromal/perivascular than in tumor-epithelial models (95% CI 1.34–1.71; P < 0.001) (Figure 6B). All six individual Program A effects were positive, with standardized beta coefficients ranging from 1.29 for BGN to 1.97 for COL3A1 after within-program FDR correction (Figure 6C).

**FIGURE 6:**
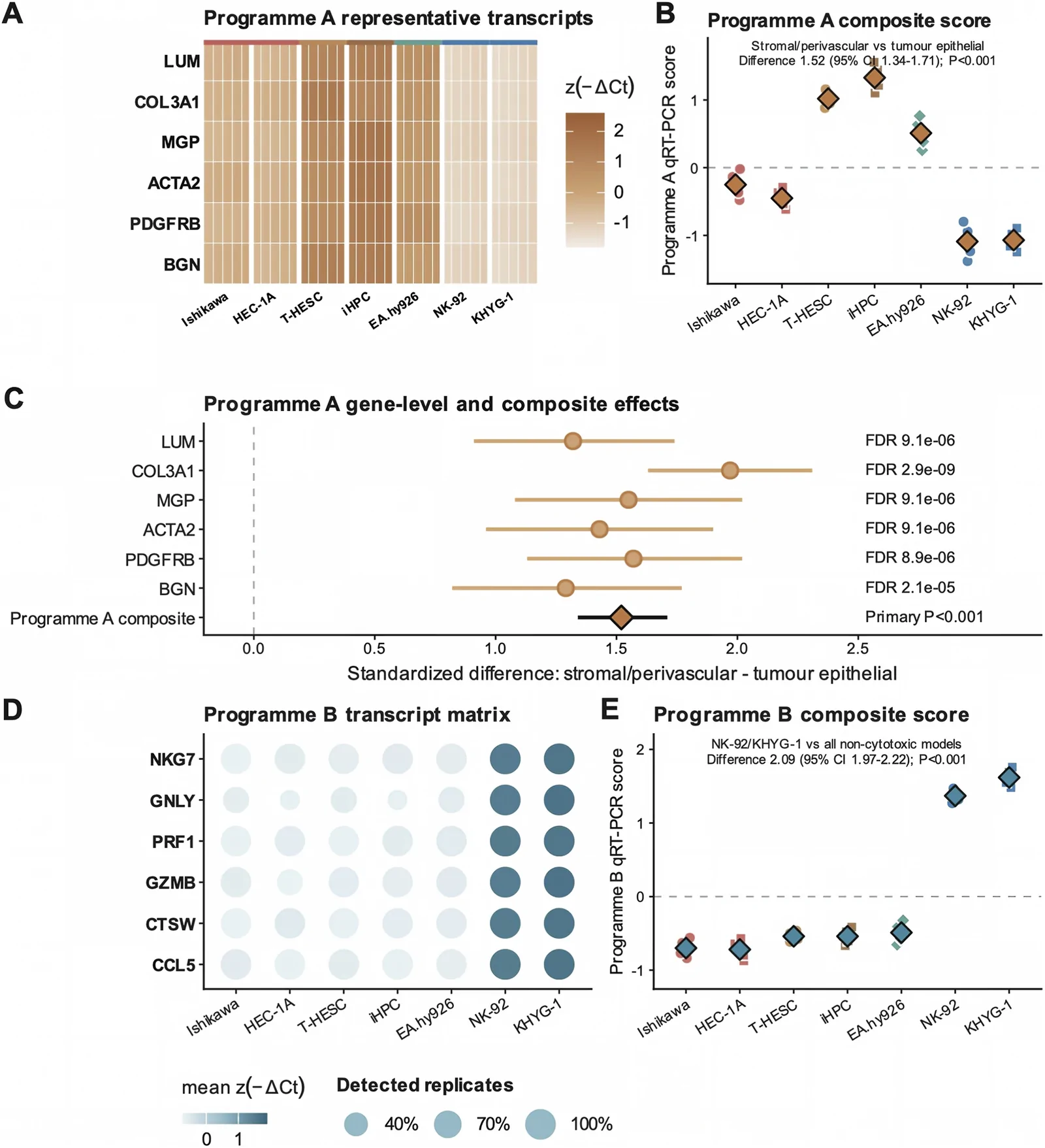
Orthogonal qRT-PCR characterization of Program A and Program B across ATCC cell models. **(A)** Row-standardized −Delta Ct heatmap of six Program A representative genes across tumor-epithelial, stromal, perivascular/smooth-muscle, endothelial, and cytotoxic-lymphocyte models. **(B)** Unweighted Program A qRT-PCR composite scores. **(C)** Stromal/perivascular-versus-tumor-epithelial effects for individual Program A genes and the composite score. **(D)** Row-standardized expression matrix of six Program B genes. **(E)** Program B composite scores in cytotoxic versus non-cytotoxic models. Points represent independent culture experiments, and summary estimates are shown with 95% confidence intervals. Gene-level P-values were adjusted separately within Program A and Program B using the Benjamini–Hochberg method. The experiment assessed cellular localization of the frozen programs and did not directly examine PPC.

NKG7, GNLY, PRF1, GZMB, CTSW, and CCL5 were markedly enriched in NK-92 and NK-92MI cells relative to the five non-cytotoxic models (Figure 6D). The composite Program B qRT-PCR score was 2.09 standardized units higher in the cytotoxic-lymphocyte models (95% CI = 1.97–2.22; P < 0.001) (Figure 6E). Together, the qRT-PCR results provided orthogonal experimental support for the broad stromal–vascular localization of Program A and the cytotoxic-lymphocyte localization of Program B. Because the commercial cell models did not carry PPC labels, the experiment evaluated cellular localization rather than the PPC association itself.

## 4 Discussion

This study places PPC within a deliberately constrained multimodal framework. In TCGA-UCEC, PPC-positive tumors showed coordinated stromal/mesenchymal-high and cytotoxic/antigen-presentation-low ranked transcriptional directions, despite the absence of any genome-wide single-gene result after FDR correction. The strongest clinical finding was the persistent association of PPC with shorter PFI, OS, and DSS. The strongest contextual finding was that Program A arose broadly from stromal–vascular compartments, whereas Program B arose from cytotoxic lymphocytes. The most important negative findings were equally informative: Program C did not show consistent multimodal support, Program A did not retain within-compartment motif associations after cell-identity adjustment, one spatial section did not support cytotoxic exclusion or hypoxia/TGF-beta coupling, and tumor CD274 transcript abundance neither increased with PPC nor credibly explained PPC-associated risk.

The clinical association of PPC is consistent with prior retrospective cohorts and meta-analyses (Pergialiotis et al., 2024; Fegus et al., 2024; Seagle et al., 2018; Kanno et al., 2022). Our results extend that literature by linking PPC to ranked tumor transcriptional directions, rather than to a newly constructed prognostic score. The PPC-positive group was substantially enriched for serous histology, high grade, advanced stage, residual disease, and copy-number-high tumors. This pattern suggests that PPC is partly a marker of aggressive tumor biology and anatomical burden. The persistence of the outcome association after adjustment does not prove that free malignant cells independently drive recurrence; unmeasured dissemination, treatment selection, and center-specific sampling remain plausible explanations.

The absence of single-gene FDR significance is central to interpreting the transcriptome. Strong GSEA results can arise when many genes move coherently but modestly along a ranked list, even when no individual gene crosses a stringent multiplicity threshold. This was the case for EMT, hypoxia, TGF-beta, and angiogenesis in the positive direction and for cytotoxic, T-cell-inflamed, and antigen-presentation programs in the negative direction. Accordingly, the data support coordinated bulk transcriptional states, not a PPC-specific hub gene or a discrete mechanistic pathway. The negative candidate screen reinforced this boundary: no leading-edge gene passed the complete sequence of PPC association, robustness, network membership, and outcome criteria.

Multimodal localization helped separate cell identity from within-cell regulation. Program A was reproducibly stromal at the broad-compartment level, but the highest fine source varied between pericyte/vascular smooth-muscle cells and fibroblast/CAF cells. This heterogeneity is biologically plausible for an ECM/mesenchymal/vascular gene set and argues against relabeling the program as a single-cell-type signature. Program B showed greater consistency: both scRNA datasets placed it in NK/CD8 compartments, scATAC GeneActivity localized it similarly, and residualized RNA-ATAC concordance remained positive after controlling cell type and patient. Program C, in contrast, showed dataset-dependent source localization, an opposite expected-source GeneActivity contrast and weak residual concordance. Treating all three programs as equally validated would therefore overstate the evidence.

The scATAC results also illustrate why cross-cell-type motif correlations require adjustment. SMAD, AP-1, and TEAD accessibility initially tracked Program A, but these associations nearly vanished after accounting for cell identity and patient. The raw signal was therefore largely a contrast between stromal and non-stromal compartments, rather than evidence that the motif families directly regulate Program A within a shared cell population. This distinction is methodologically important because single-cell atlases can generate apparently strong regulatory relationships whenever both variables encode lineage. Residual analyses and within-compartment contrasts should precede mechanistic interpretation.

The spatial analysis yielded a similar correction to an intuitive but unsupported narrative. Program A was strongly organized within the UCEC3 section and co-occurred with stromal–vascular composition in unadjusted maps. Raw correlation with CD8/NK was positive rather than negative. Once the closed-sum nature of RCTD weights and technical composition were addressed, the relationship approached zero. Thus, a bulk cytotoxic-low direction did not translate into spatial cytotoxic exclusion in the available tissue. Likewise, Program A did not show stable adjusted coupling with hypoxia or TGF-beta. Because only one UCEC spatial section was available and the residual model was collinearity-limited, these analyses do not establish absence across endometrial cancer; they show that the current section does not support those claims.

The CD274 result narrowed the original immunological hypothesis. PPC-positive tumors did not show higher tumor CD274 transcript abundance in any principal or sensitivity model, and CD274 was not associated with survival after adjustment. The PPC-by-CD274 interactions were also uncertain. These observations do not address PD-L1 protein expression, tumor-proportion score, combined positive score, or response to PD-1/PD-L1 blockade. They do, however, argue against a simple model in which PPC-related risk is explained by higher bulk-tumor CD274 transcription.

The ATCC-based qRT-PCR experiment provided an independent cellular assay for the program assignments. Program A transcripts were consistently higher in the stromal and perivascular models, whereas Program B transcripts were strongly enriched in the two NK models. These findings agreed with the broad cellular localization observed in the single-cell and GeneActivity analyses and reduced the likelihood that the compartment assignments were specific to a single public dataset. The experiment did not use PPC-labeled patient material and therefore did not evaluate whether PPC caused or increased either program.

Several limitations should be emphasized. First, TCGA-UCEC is the only PPC-labeled cohort; no external dataset contains both comparable PPC annotation and transcriptomic data. Second, PPC-positive patients were fewer and had markedly higher-risk clinicopathological features, leaving room for residual confounding. Third, the external single-cell and chromatin datasets lacked PPC labels and therefore contextualized rather than validated PPC effects. Fourth, GSE173682 included five endometrial-tumor patients, and GSE203612 included only three; some source contrasts were necessarily descriptive. Fifth, the spatial analysis was based on one section without pathologist-frozen regions. Sixth, the reference-sensitive abundance/state decomposition could not robustly separate cell abundance from cell-intrinsic expression and was therefore not included in the primary analysis or interpretation. Finally, the qRT-PCR experiments used established commercial cell models and confirmed compartmental expression patterns, but they did not recapitulate the multicellular tumor microenvironment or directly evaluate PPC.

Future studies should prioritize a second patient-level PPC cohort, standardized cytology sampling, harmonized molecular classification, and collection of tumor tissue suitable for RNA profiling, digital pathology, and spatial analysis. Stronger biological inference will require PPC-labeled patient-derived tissues or lavage specimens, repeated spatial sections, and functional assays designed around prospectively defined hypotheses, rather than *post hoc* single-gene narratives.

## 5 Conclusion

PPC-positive endometrial cancers in TCGA-UCEC were associated with adverse outcomes and coordinated stromal/mesenchymal-high and cytotoxic/antigen-presentation-low ranked bulk transcriptional directions. Single-cell, chromatin-accessibility, and qRT-PCR data localized Program A broadly to stromal–vascular compartments and Program B to cytotoxic lymphocytes. Program A was spatially organized in one UCEC section, but composition-adjusted analyses did not provide evidence of cytotoxic exclusion or stable hypoxia/TGF-beta coupling. Program C was not consistently supported. Tumor CD274 transcript abundance neither increased with PPC nor credibly explained or modified the PPC-associated clinical risk. These findings provide a multimodal cellular context for PPC-associated transcriptional patterns without establishing a causal mechanism.

## Data Availability

TCGA-UCEC data are available through the NCI Genomic Data Commons. GSE173682 and GSE203612 are available through the NCBI Gene Expression Omnibus. Analysis scripts, frozen manifests, audit tables, derived results, and qRT-PCR data are available from the corresponding author upon reasonable request.
